# Functional Trade-Offs of Regulatory Mechanisms in the Management of Body Energy, Frontal Plane Angular Momentum and Mediolateral Margin of Stability During Hole Negotiation Gait

**DOI:** 10.1007/s10439-025-03835-7

**Published:** 2025-09-08

**Authors:** Adamantios Arampatzis, Maria-Elissavet Nikolaidou, Christos Theodorakis, Morteza Ghasemi, Falk Mersmann, Sebastian Bohm

**Affiliations:** 1 Department of Training and Movement Sciences, Humboldt-Universität zu Berlin, Philippstr. 13, Haus 11, 10115 Berlin, Germany; 2 Berlin School of Movement Science, Berlin, Germany; 3 Division of Sport Medicine and Biology of Exercise, Faculty of Physical Education and Sport Science, National and Kapodistrian University of Athens, Athens, Greece

**Keywords:** Challenging locomotion, Muscle activation, Leg posture, Joint kinematics, Uneven terrain

## Abstract

The functional interaction of regulatory mechanisms that manage total centre of mass (CoM) energy, frontal plane whole-body angular momentum and mediolateral margin of stability (MoS) during hole negotiation gait was investigated. Joint kinematics, leg posture, total CoM energy, frontal plane whole-body angular momentum, mediolateral MoS and muscle activation patterns of seven bilateral lower leg muscles were assessed in 18 participants. During hole negotiation, we found an increase in the peak-to-peak range of total CoM energy and frontal plane whole-body angular momentum during the preparation, hole and recovery steps, and a decrease in mediolateral MoS at touch-down during the preparation and hole steps compared to level walking, providing evidence of an increased challenge in stability control. Anticipatory adjustments in CoM trajectories, joint kinematics and muscle activation patterns regulated mechanisms that primarily supported the management of total CoM energy at the expense of whole-body angular momentum in the frontal plane. We identified an anticipatory foot placement strategy during the step in the hole that significantly reduced the moment arm of the vertical ground reaction force (*p* = 0.011, *d* = 0.81), thereby favouring the control of frontal plane whole-body angular momentum. Conversely, this foot placement strategy significantly reduced (*p* < 0.001, *d* = 1.05) the mediolateral MoS. The mutual influence between the regulatory mechanisms that control total CoM energy, frontal plane angular momentum and mediolateral MoS represent trade-offs rooted in the nature of the hole negotiation gait and demonstrate the challenge of moving on uneven terrain.

## Introduction

Moving on uneven terrain is linked to external mechanical perturbations, heightened gait instability and elevated risk of falling [1–3]. On uneven terrain, managing body energy, whole-body angular momentum and stability during walking becomes more challenging [4–8]. As a consequence, the resultant joint moments, joint mechanical power, range of whole-body angular momentum and electromyographic (EMG) activity in the muscles of the lower extremities increase on uneven terrain [5, 6, 9]. While in the sagittal plane, the natural swing of the legs simplifies dynamic stability control during locomotion, active control of body stability is required in the frontal plane [10, 11]. The range of whole-body angular momentum in the frontal plane increases during stair negotiation [12, 13], uphill and downhill walking [4] and mediolateral gait perturbations [14, 15], indicating increased difficulty in maintaining stability in the frontal plane during walking on uneven terrain and in the presence of perturbations.

Negotiating a hole as a specific movement task for moving on uneven terrain is common in everyday life and can lead to falls if the step is incorrectly placed on the ground [1, 2]. Biological systems are able to prepare an expectation of sensory responses through experience and visual guidance to reduce locomotor disturbances [16–18]. Experience-based and visually guided anticipatory adjustments provide the opportunity to proactively plan movement initiation and modify subsequent responses, facilitating robust and stable gait [19–22]. When stepping down, adjustments in leg retraction and the trajectory of the body's centre of mass (CoM), fine-tuning of lower leg muscle activation, switching from heel to toe landing are key mechanisms that affect stance dynamics, muscle work loops and body energy management in humans [19, 23–25] and other animals [26–28]. A lowering of the body's CoM as an anticipatory adjustment begins already in the step before touch-down in the hole (i.e. preparation step) and continues in the first part of the subsequent stance (hole step), showing a greater range of motion of the vertical trajectory of the CoM during hole negotiation compared to level walking [24, 29]. The greater vertical range of motion of the CoM, i.e. greater range of the potential energy of the body, suggests an increased challenge to manage total CoM energy during hole negotiation. Indeed, higher EMG-activity in the muscles of both the ipsilateral and contralateral leg has been reported during hole negotiation gait compared to level walking [19, 29, 30].

The increased EMG-activity, and possibly the increased force generation by the leg muscles, may help to manage the total CoM energy that is required to negotiate the hole. On the other hand, increased force generation in the triceps surae and vasti muscles of the stance leg increases whole-body angular momentum towards the contralateral leg [31]. Therefore, the increased activation of the leg extensor muscles during hole negotiation gait could make the management of whole-body angular momentum in the frontal plane more difficult, challenging movement stability [13, 32, 33]. An increase in the range of whole-body angular momentum in the frontal plane due to increased activation of the triceps surae and vasti muscles during hole negotiation would indicate a regulation of body CoM energy at the expense of whole-body angular momentum. This possible scenario could suggest a trade-off in the mechanisms that manage total CoM energy and frontal plane angular momentum. Mediolateral adjustments in leg posture and muscle activation patterns affect the mediolateral base of support (BoS) and the mediolateral moment arm from the body's CoM to the vertical ground reaction force vector, and thus influences the regulation of frontal plane whole-body angular momentum and mediolateral margin of stability (MoS) [34–36]. An anticipatory modification of the mediolateral leg angle at touch-down during hole negotiation, i.e. closer to the vertical axis through a foot placement strategy [37] can reduce the mediolateral moment arm of the vertical ground reaction force, favouring the regulation of the frontal plane angular momentum [33]. On the other hand, this adjustment results in a position of the body CoM closer to the mediolateral limits of the BoS, thus compromising the stability state of the body by reducing the MoS in the mediolateral direction [35]. This scenario could again suggest a trade-off in the mechanisms that manage the rotational, i.e. frontal plane angular momentum, and translational, i.e. mediolateral MoS, behaviour of the body during hole negotiation gait. In this context, there is no information on the functional interaction and diversity of mechanisms that regulate the management of total CoM energy, frontal plane angular momentum and mediolateral MoS to maintain a stable gait during hole negotiation. Understanding the neuromuscular and biomechanical mechanisms that control the translational and rotational behaviour of the whole-body during movement on uneven terrain will provide important knowledge for effective stability control and fall prevention.

The purpose of the current study was to investigate how humans regulate total CoM energy, whole-body angular momentum in the frontal plane and mediolateral MoS to maintain balance during hole negotiation gait. In particular, the functional interaction and diversity of regulatory mechanisms such as leg posture, angular joint trajectories and muscle activation patterns for managing total CoM energy, frontal plane angular momentum and mediolateral MoS were investigated. During hole negotiation gait, we hypothesized (a) an increased peak-to-peak range in the frontal plane whole-body angular momentum and total CoM energy, as well as a decrease in the mediolateral MoS, thus increasing the challenge to manage total CoM energy, frontal plane angular momentum and mediolateral MoS compared to level walking, (b) anticipatory adjustments of regulatory mechanisms that control the management of total CoM energy, frontal plane angular momentum and mediolateral MoS and (c) trade-offs on the mechanisms regulating total CoM energy, frontal plane angular momentum and mediolateral MoS.

## Methods

### Hole Negotiation Paradigm

**Fig. 1:**
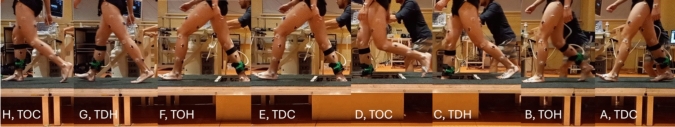
Characteristic time points during the hole negotiation gait. Touch-down of the contralateral (left) leg in the preparation step (**A**, TDC), take-off of the hole (right) leg in the preparation step (**B**, TOH), touch-down of the hole leg in the hole (**C**, TDH), take-off of the contralateral leg in the hole step (**D**, TOC), touch-down of the contralateral leg in the recovery step (**E**, TDC), take-off of the hole leg from the hole (**F**, TOH), touch-down of the hole leg in the recovery step (**G**, TDH) and take-off of the contralateral leg in the recovery step (**H**, TOC)

The hole negotiation experiments were conducted on an 18 m long custom-built gangway with a hole (15 cm deep, 70 cm long and 46 cm wide) located in the second half of the gangway. Eighteen participants (5 female and 13 male, age 24.8 ± 5.4 years, body mass 75.2 ± 9.7 kg, height 176.5 ± 7.8 cm) gave written informed consent in accordance with the Declaration of Helsinki and participated in the study. The study was approved by the Ethics Committee of the Humboldt-Universität zu Berlin (HU-KSBF-EK_2022_0032). The starting position at the beginning of the gangway was carefully adjusted through several familiarization trials to ensure that participants negotiated the hole with their right leg at their preferred walking speed (1.3 ± 0.1 m/s, Fig. 1). Throughout the manuscript, the right leg was defined as the hole leg and the left leg as the contralateral leg. After adjusting the starting position, participants performed three level walking trials and continued with five hole negotiation gait trials. We analysed the first level walking trial and the 5th hole negotiation gait trial. We analysed the 5th negotiation trial to consider a visually guided, experience-based hole negotiation gait in our study. A harness system was used to ensure the safety of the participants as they walked freely along the gangway.

### Body Kinematics and Electromyography

An infrared motion capture system (Vicon Nexus, version 2.12, Vicon Motion Systems, Oxford, UK) with 19 cameras operating at 250 Hz was used to measure the three-dimensional coordinates of 22 markers attached to anatomically referenced locations on the second metatarsal, tuber calcaneus, lateral malleolus, lateral epicondyle, greater trochanter, acromion, elbow and wrist bilaterally, sacrum, 7th cervical vertebra and cranial bone (2 anterior and 2 posterior). The three-dimensional coordinates of the markers were filtered using a fourth order low-pass Butterworth filter with zero phase shift and a cut-off frequency of 12 Hz. The method of Maiwald et al. [38] was used to determine the touch-down and take-off of the foot. The characteristic maximum of the vertical acceleration of the second metatarsal was used to define take-off, while characteristic maxima of the vertical acceleration of the calcaneus or the second metatarsal marker were used to define touch-down during rear-foot or fore-foot contact. The anteroposterior distance of the calcaneus markers between the two legs at the beginning of the double contact phase was defined as step length, and the mediolateral distance of the second metatarsal markers between the two legs as step width. We calculated the joint angles as the angle between two vectors in 3D space. The ankle joint angle was defined as the angle formed by the vectors connecting the second metatarsal and the lateral malleolus, and the lateral malleolus and the lateral epicondyle. The knee joint angle was defined as the angle formed by the vectors connecting the lateral malleolus and the lateral epicondyle, and the lateral epicondyle and the greater trochanter. The hip joint angle will be referred to as the angle formed by the vectors connecting the lateral epicondyle and the greater trochanter, and the greater trochanter and the 7th cervical vertebra. The three joint angles were calculated with respect to a neutral, quiet stance position (0° for the ankle and 180° for the knee and hip joint angles). Plantarflexion of the ankle is indicated by positive values and dorsiflexion of the ankle by negative values, while values less than 180° for the knee and hip angles represent a flexed joint position. The angle between the vertical axis of the global coordinate system and the line crossing the CoM of the foot and the CoM of the body, calculated in the frontal and sagittal planes, defines the mediolateral and anteroposterior leg angles, with lower values closer to the vertical axis. The mediolateral moment arm of the vertical ground reaction force vector was calculated according to Neptune and Vistamehr [33] as the mediolateral distance between the CoM of the body and the CoM of the foot using the coordinates of the mediolateral axis of the global coordinate system. The stance phases of three consecutive steps were included in the analysis (preparation: touch-down of the contralateral leg before touch-down in the hole and until take-off, hole: touch-down of the hole leg and until take-off and recovery: touch-down of the contralateral leg after touch-down in the hole and until take-off).

Total CoM energy was calculated as the sum of potential and kinetic CoM energy (Eq. 1):

1 $${E}_{\text{CoM}}=mg{h}_{\text{CoM}}+ \frac{1}{2}m{{{\varvec{v}}}_{\mathbf{b}\mathbf{o}\mathbf{d}\mathbf{y}}^{\mathbf{C}\mathbf{o}\mathbf{M}}}^{2}$$

where *m* is the mass of the body (kg), *g* is the acceleration of gravity (m/s^2^), ${h}_{\text{CoM}}$ is the height of the CoM (m) and ${{\varvec{v}}}_{\mathbf{b}\mathbf{o}\mathbf{d}\mathbf{y}}^{\mathbf{C}\mathbf{o}\mathbf{M}}$ is the velocity vector of the body CoM (m/s). The masses of the segments and their positions within each segment to calculate the CoM were taken from Dempster [39].

The angular momentum of the whole-body around the CoM has been calculated as follows (Eq. 2):

2 $$H= \sum_{i=1}^{n}\left[\left({r}_{i}^{\text{CoM}}- {r}_{\text{body}}^{\text{CoM}}\right) \times {m}_{i}\left({v}_{i}^{\text{CoM}}- {v}_{\text{body}}^{\text{CoM}}\right)+ {I}_{i}{\omega }_{i}\right]$$

where *H* is the whole-body angular momentum $(kg\cdot {m}^{2}/s)$, ${r}_{i}^{\text{CoM}}$ is the position vector of the ${i}_{\text{th}}$ segment’s CoM $(m)$, ${r}_{\text{body}}^{\text{CoM}}$ is the position vector of the whole-body CoM $(m)$, ${{\varvec{m}}}_{{\varvec{i}}}$ is the mass $(kg)$ of the ${i}_{\text{th}}$ segment, ${v}_{i}^{\text{CoM}}$ is the velocity vector of the ${i}_{\text{th}}$ segment’s CoM $(m/s)$, ${I}_{i}$ is the moment of inertia $(kg\cdot {m}^{2})$ of the ${i}_{\text{th}}$ segment, ${\omega }_{i}$ is the angular velocity vector of the ${i}_{\text{th}}$ segment $(\text{rad}/s)$ and *n* is the number of the body segments (*n* = 12). The whole-body angular momentum was determined in the frontal plane. From the frontal plane whole-body angular momentum and total CoM energy curves, we examined the peak-to-peak range of frontal plane angular momentum and the peak-to-peak range of total CoM energy during the stance phase of the preparation step, the stance phase of the hole step and the stance phase of the recovery step. Furthermore, we calculated the rate of frontal plane angular momentum during the single-leg stance phase, which is equal to the external moment of the ground reaction force vector around the CoM in the frontal plane and rotates the body towards the contralateral leg.

The assessment of the stability state of the human body in the mediolateral direction was performed by calculating the MoS using the extrapolated CoM approach presented by Hof et al. [40] as follows (Eq. 3):

3 $$b={U}_{\text{max}}-\left({P}_{\text{CoM}}+ \frac{{v}_{\text{CoM}}}{\sqrt{\frac{g}{l}} }\right)$$

where $b$ is the MoS (m), ${U}_{\text{max}}$ is the mediolateral boundary of the BoS (m), ${P}_{\text{CoM}}$ is the mediolateral component of the projection of the body's CoM onto the ground (m), ${v}_{\text{CoM}}$ is the mediolateral velocity of the body's CoM (m/s), $g$ is the acceleration of gravity (m/s^2^) and $l$ is the distance between the body's CoM and the centre of the ankle joint (m). The term in brackets is the extrapolated centre of mass (m).

Using a wireless EMG system (myon AG, Schwarzenberg, Switzerland), the EMG-activity of the soleus (SOL), gastrocnemius medialis (GM), gastrocnemius lateralis (GL), tibialis anterior (TA), vastus lateralis (VL), vastus medialis (VM) and biceps femoris (BF) muscles was measured bilaterally at 2000 Hz synchronized with the motion capture system. The measured EMG-signal was filtered using a fourth order Butterworth high pass filter with a cut-off frequency of 20 Hz, then full wave rectified and finally low pass filtered with a cut-off frequency of 20 Hz. The maximum EMG-activity obtained during a maximal voluntary fixed-end plantarflexion for SOL, GM, GL, dorsiflexion for TA, knee extension for VL, VM and knee flexion for BF contractions was used to normalize the EMG values for each participant. The first-order differential equation proposed by Zajac [41] was used to estimate the muscle activation ($\widehat{\alpha }$) of the studied muscles from the measured normalized EMG-activity ($\widehat{u}$) of the muscles as follows (Eq. 4):

4 $$\frac{d \widehat{\alpha }\left(t\right)}{d t} +\left[\frac{1}{{\tau }_{\text{act}}} \cdot \left(\beta + \left[1-\beta \right] \widehat{u}\left(t\right)\right)\right]\cdot \widehat{\alpha }\left(t\right)=\left(\frac{1}{{\tau }_{\text{act}}} \right)\cdot \widehat{u}\left(t\right)$$

The specific activation time constant (τ_act_) and the ratio of activation to deactivation time constant (β) of slow and fast twitch fibres [42] are determined considering the reported fibre type distribution for SOL (slow: 78%, fast: 22%), GM, GL (slow: 50%, fast: 50%), TA (slow: 60%, fast: 40%), VL and VM (slow: 38%, fast: 62%) and BF (slow: 63%, fast: 37%) [43–45]. An average weighted activation of the triceps surae muscles (SOL, GM, GL) and the vasti muscles (VL, VM) according to their volume ratios was also calculated. The values reported by Albracht et al. [46] for the triceps surae muscles (SOL: 0.52, GM: 0.32, GL: 0.16) and by Mersmann et al. [47] for the vasti muscles (VL: 0.57, VM: 0.43) were used.

### Statistics

A linear mixed model was used to test the main effect of locomotor condition (level walking vs. hole negotiation) on the outcomes investigated, i.e. temporal and spatial parameters, joint kinematics, leg posture, horizontal CoM velocity, CoM energy, frontal plane whole-body angular momentum, mediolateral MoS, muscle activation, and was applied separately to the three steps (preparation, hole, recovery). Participants were considered as a random effect in the statistics, while locomotor condition was considered as a fixed effect. A linear mixed model was further used to examine the association between the weighted activation of the triceps surae and vasti muscles and the range of total CoM energy and frontal plane angular momentum during the stance phase of the preparation, hole and recovery steps. The activation of the lower leg muscles can influence the magnitude and direction of the ground reaction force vector, thereby affecting the range of the whole-body angular momentum and the total CoM energy. Since the magnitude of the ground reaction forces primarily affects changes in the total CoM energy, we focused on the total CoM energy in our calculations. It should be noted that ground reaction forces may also affect the rotational energy of the body, which is not considered in the calculation of total CoM energy. Nevertheless, the body's rotational energy is small compared to its translational and potential energy due to its low moment of inertia and angular velocity. One advantage of linear mixed models is that they are robust against violations of the normality assumption [48], which was not met for maximum activation of the VL and BF in the preparation step, step length and maximum activation of the GL, TA, VM and VL in the hole step, and MoS and maximum activation of the GM, GL and VL in the recovery step, according to the Shapiro–Wilk test applied to the normalized residuals. The statistical analyses were performed using R version 4.0.1 (R Foundation for Statistical Computing, Vienna, Austria). Statistical parametric mapping (SPM) [49] was also performed to compare continuous variables (i.e. time-normalized vectors) between level and hole negotiation trials. Ankle, knee and hip joint angles, CoM energies, whole-body angular momentum in the frontal plane and muscle activation during the three steps studied were the continuous time-normalized variables. The significance level was set at *α* = 0.05 for all test comparisons. In addition, Cohen's effect size (*d*) was calculated to assess the strength of potential differences between the variables investigated. Values of *d* < 0.2 indicate small effect sizes, 0.2 ≤ *d* < 0.8 indicate medium effect sizes and *d* ≥ 0.8 indicate large effect sizes [50].

## Results

**Fig. 2:**
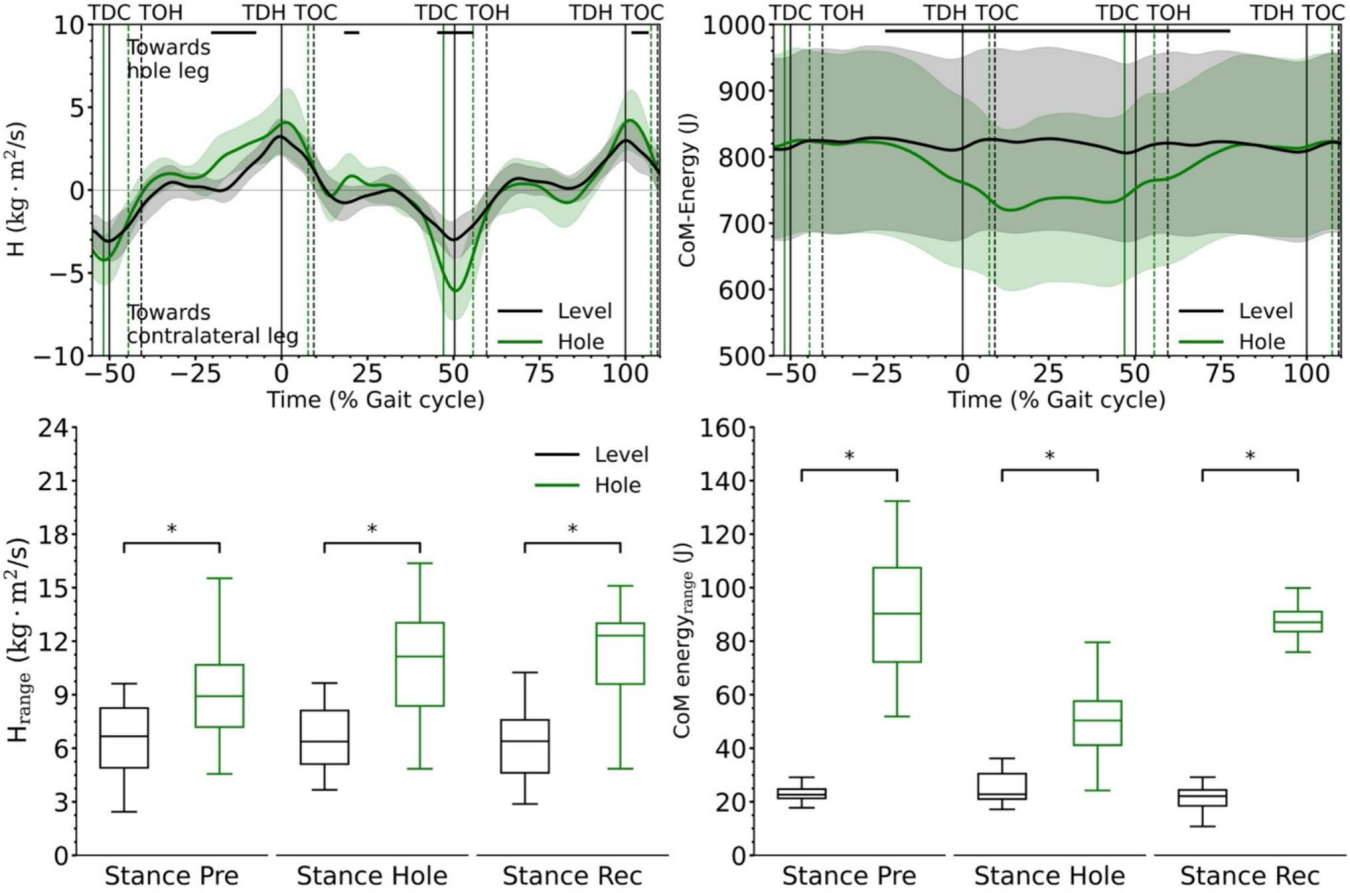
Frontal plane whole-body angular momentum (H) and total centre of mass (CoM) energy during level and hole negotiation walking. Positive values in the whole-body angular momentum indicate rotation towards the hole (right) leg and negative values towards the contralateral (left) leg. The curves and shaded areas represent mean ± standard deviation. The horizontal axis is normalized to the gait cycle before touch-down in the hole (negative percentages) and to the gait cycle after touch-down in the hole (positive percentages). The vertical solid lines show the touch-down of the contralateral and the hole leg and the vertical dashed lines show the take-off. The horizontal black lines in the upper part of the figures define the areas of significant differences between level and hole negotiation (*p* < 0.05) based on the SPM analysis. *TDC* touch-down of the contralateral leg, *TOH* take-off of the hole leg, *TDH* touch-down of the hole leg, *TOC* take-off of the contralateral leg. The boxplots show the peak-to-peak range of frontal plane angular momentum and peak-to-peak range of total CoM energy during the stance phase of the preparation step (Stance Pre), the stance phase of the hole step (Stance Hole) and the stance phase of the recovery step (Stance Rec). *Statistically significant (*p* < 0.05) differences

The peak-to-peak range of frontal plane angular momentum and the range of total CoM energy during the stance phase were significantly greater in hole negotiation than in level walking for all three steps examined (preparation: *p* < 0.001, *d* = 1.01 and *p* < 0.001, *d* = 5.50; hole: *p* < 0.001, *d* = 1.61 and *p* < 0.001, *d* = 2.81; recovery: *p* < 0.001, *d* = 1.84 and *p* < 0.001, *d* = 5.49 for angular momentum and CoM energy, respectively, Fig. 2). During hole negotiation, the range of frontal plane angular momentum during the single-leg stance phase was also significantly greater in all three steps investigated (preparation: *p* = 0.027, *d* = 0.65; hole: *p* < 0.001, *d* = 1.07; recovery: *p* < 0.001, *d* = 1.33, Table 1). The rate of frontal plane angular momentum during the single-leg stance phase was significantly greater during the hole (*p* < 0.001, *d* = 1.09) and recovery steps (*p* < 0.001, *d* = 1.08) of the hole negotiation (Table 1). In the hole negotiation, the mean mediolateral moment arm of the vertical ground reaction force vector during the stance phase was significantly lower in the preparation (*p* = 0.030, *d* = 0.59) and hole (*p* = 0.011, *d* = 0.81) steps, but did not differ between the two gait conditions in the recovery step (*p* = 0.329, *d* = 0.27, Table 1). Step length was greater in the hole (*p* < 0.001, *d* = 0.91) and recovery (*p* < 0.001, *d* = 0.85) steps and step width was smaller (*p* = 0.006, *d* = 0.85) in the hole step during hole negotiation compared to level walking (Table 1). Significant (*p* = 0.002, *d* = 0.73) changes in stance phase duration were found only in the recovery step (increase) during hole negotiation (Table 1). The average anteroposterior CoM velocity during the stance phase of the preparation, hole and recovery steps did not differ between level walking and hole negotiation.

**Table 1:** Duration of the stance phase (Stance time), step length, step width, average anteroposterior (A/P) centre of mass (CoM) velocity during the stance phase, mediolateral (M/L) and A/P leg angle at touch-down (TD), average mediolateral moment arm during stance, range and rate of whole-body angular momentum (H) during the single stance phase and mediolateral margin of stability (MoS) at touchdown during the preparation, in the hole and recovery steps in level and hole negotiation gait (mean ± standard deviation)

Variables	Preparation step		Step in the hole		Recovery step	
	Level walking	Hole negotiation	Level walking	Hole negotiation	Level walking	Hole negotiation
Stance time (ms)	632 ± 45	650 ± 58	642 ± 47	622 ± 42	636 ± 47	674 ± 57*
Step length (cm)	71.3 ± 4.9	70.3 ± 6.5	67.7 ± 5.1	73.9 ± 8.4*	70.6 ± 5.0	75.0 ± 5.2*
Step width (cm)	18.5 ± 1.9	18.8 ± 2.4	19.6 ± 2.4	17.3 ± 2.8*	19.1 ± 3.2	19.8 ± 4.0
CoM Velocity_A/P_ (m/s)	1.33 ± 0.12	1.33 ± 0.14	1.32 ± 0.13	1.33 ± 0.15	1.31 ± 0.11	1.32 ± 0.14
Leg angle_M/L_ at TD (°)	7.7 ± 1.3	6.7 ± 1.4*	6.4 ± 1.5	4.3 ± 1.4*	6.9 ± 2.0	8.8 ± 3.1*
Leg angle_A/P_ at TD (°)	31.7 ± 2.7	31.4 ± 4.5	33.1 ± 2.6	26.1 ± 2.9*	30.1 ± 2.7	37.5 ± 4.8*
Moment arm_M/L_ (cm)	5.4 ± 1.1	4.7 ± 1.4*	4.4 ± 1.3	3.3 ± 1.3*	4.9 ± 1.2	5.4 ± 2.3
H_range,single_ (kgm^2^/s)	4.4 ± 1.8	6.0 ± 2.3*	4.3 ± 1.8	7.3 ± 3.5*	3.9 ± 1.9	7.7 ± 3.6*
Rate H_range,single_ (Nm)	10.3 ± 4.3	12.5 ± 5.9	9.6 ± 4.1	16.5 ± 7.7*	9.0 ± 4.4	15.3 ± 6.7*
MoS_M/L_ (cm)	5.5 ± 1.4	5.0 ± 1.6*	4.5 ± 1.3	3.1 ± 1.3*	4.9 ± 1.3	4.6 ± 2.1

*Statistically significant difference to level walking (*p* < 0.05)

**Fig. 3:**
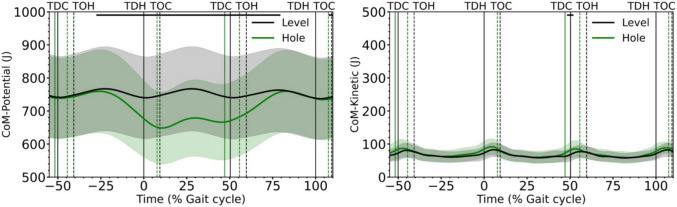
Potential and kinetic energy of the centre of mass (CoM) during level and hole negotiation walking. The curves and shaded areas represent mean ± standard deviation. The horizontal axis is normalized to the gait cycle before touch-down in the hole (negative percentages) and to the gait cycle after touch-down in the hole (positive percentages). The vertical solid lines show the touch-down of the contralateral (left) and the hole (right) leg and the vertical dashed lines show the take-off. The horizontal black lines in the upper part of the figures define the areas of significant differences between level and hole negotiation (*p* < 0.05) based on the SPM analysis. *TDC* touch-down of the contralateral leg, *TOH* take-off of the hole leg, *TDH* touch-down of the hole leg, *TOC* take-off of the contralateral leg

**Fig. 4:**
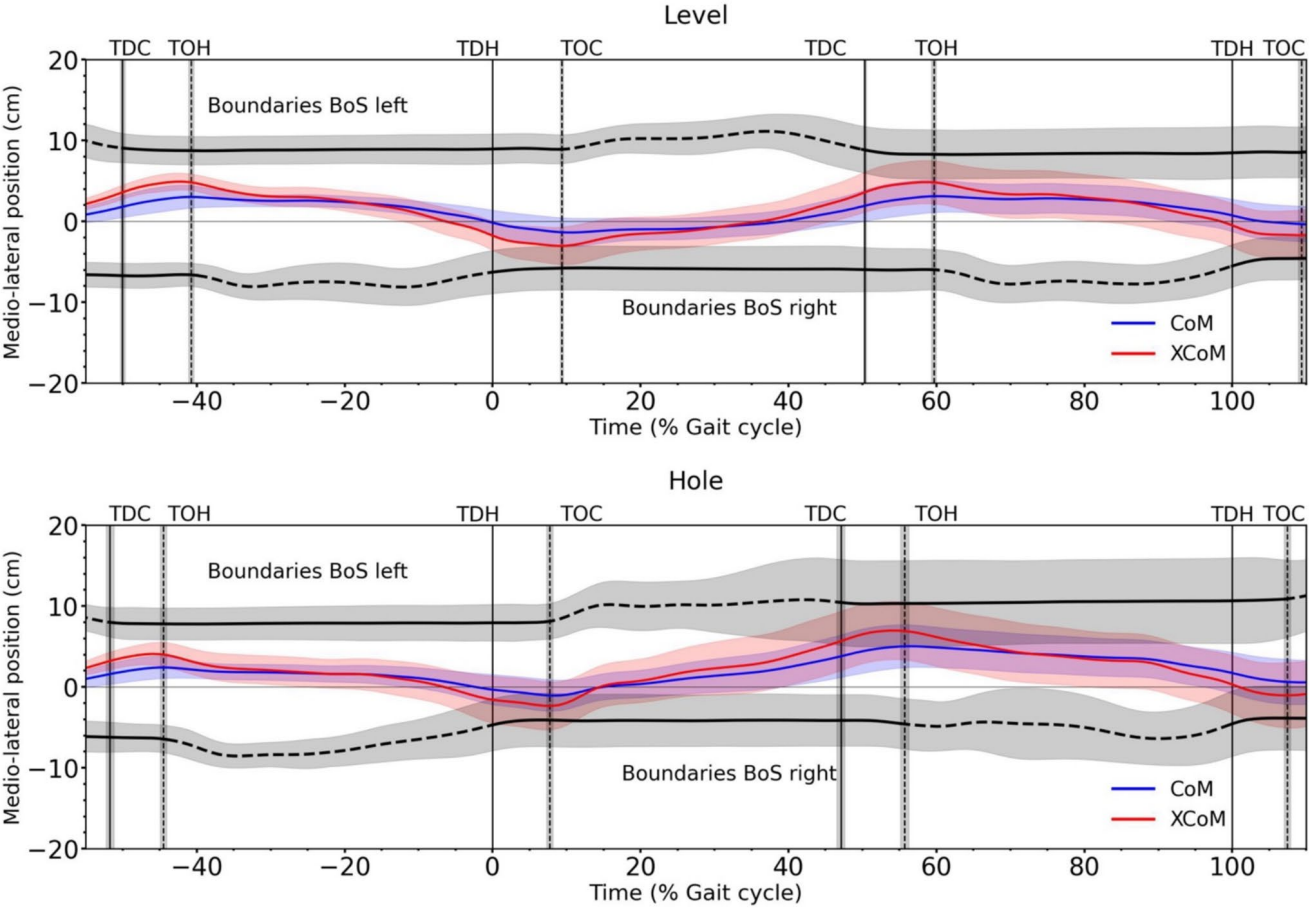
Mediolateral boundaries of the base of support (BoS), mediolateral position of the centre of mass (CoM) and mediolateral position of the extrapolated CoM (XCoM) during level (first row) and hole negotiation walking (second row). Solid lines in the boundaries of the BoS indicate that the leg is in contact with the ground, and dashed lines indicate that the leg is not in contact with the ground. The curves and shaded areas represent mean ± standard deviation. The horizontal axis is normalized to the gait cycle before touch-down in the hole (negative percentages) and to the gait cycle after touch-down in the hole (positive percentages). The vertical solid lines show the touch-down of the contralateral (left) and the hole (right) leg and the vertical dashed lines show the take-off. *TDC* touch-down of the contralateral leg, *TOH* take-off of the hole leg, *TDH* touch-down of the hole leg, *TOC* take-off of the contralateral leg

The SPM analysis showed that the total CoM energy was reduced from the middle of the preparation step and up to the beginning of the single stance of the step in the hole during the hole negotiation gait, reaching again values of the level walking in the middle of the recovery step (Fig. 2). The observed trajectory of total CoM energy is the result of similar changes in potential CoM energy, as the time course of kinetic CoM energy was not significantly different (*p* > 0.05) for most of the time between the two gait conditions (Fig. 3). The whole-body angular momentum in the frontal plane was significantly greater (*p* < 0.05) between the level and hole negotiation gaits in the second part of the stance phase of the preparation step and at the end of the stance phase of the hole and recovery steps (Fig. 2). During the hole negotiation gait, MoS in the mediolateral direction at touchdown decreased significantly in the preparation (*p* = 0.039, *d* = 0.30) and hole (*p* < 0.001, *d* = 1.05) steps, but was not different (*p* = 0.361, *d* = 0.19) in the recovery step compared to level walking (Fig. 4 and Table 1). The mediolateral and anteroposterior leg angles at touchdown were significantly lower in the hole step (*p* < 0.001, *d* = 1.37 and *p* < 0.001, *d* = 2.78) and significantly higher in the recovery step (*p* = 0.005, *d* = 0.70 and *p* < 0.001, *d* = 1.66) during the hole negotiation gait (Table 1). In the preparation step, only the mediolateral leg angle has significant lower (*p* = 0.010, *d* = 0.70) values in the hole negotiation (Table 1).

**Fig. 5:**
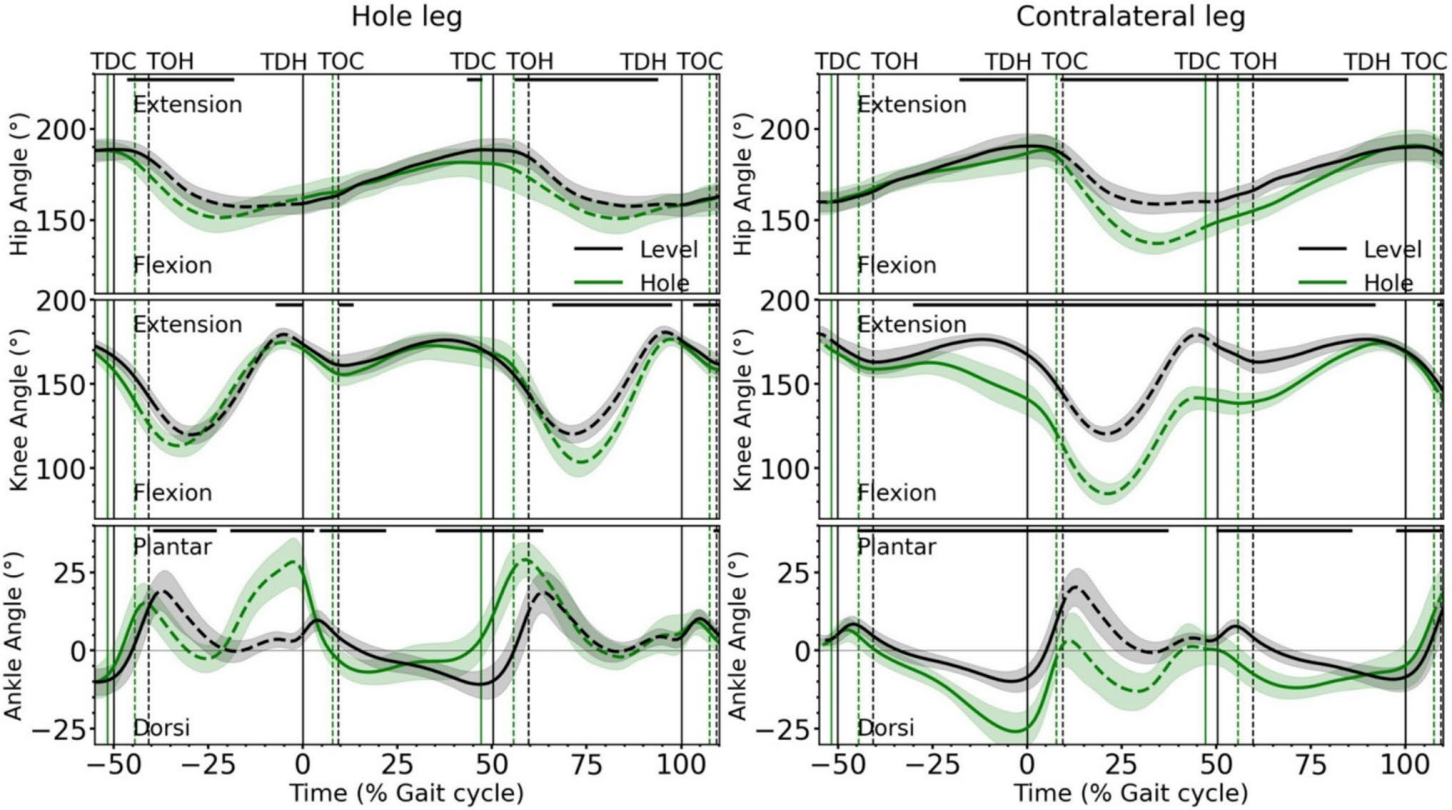
Ankle, knee and hip joint angles of the hole (right) and contralateral (left) leg during level and hole negotiation walking. Positive values for the ankle angle represent a plantarflexed joint position and negative values represent a dorsiflexed joint position. The curves and shaded areas represent mean ± standard deviation. The solid part of the curves indicates stance phase and the dashed part indicates swing phase. The horizontal axis is normalized to the gait cycle before touch-down in the hole (negative percentages) and to the gait cycle after touch-down in the hole (positive percentages). The vertical solid lines show the touch-down of the contralateral and the hole leg and the vertical dashed lines show the take-off. The horizontal black lines in the upper part of the figures define the areas of significant differences between level and hole negotiation (*p* < 0.05) based on the SPM analysis. *TDC *touch-down of the contralateral leg, *TOH* take-off of the hole leg, *TDH* touch-down of the hole leg, *TOC* take-off of the contralateral leg

Significant (*p* < 0.05) modifications in the joint kinematic patterns of both the hole and the contralateral leg started already at the beginning of the preparation step and continued until the end of the stance phase of the recovery step (Fig. 5). The ankle joint of the hole leg was more plantarflexed and the knee joint was more flexed at touch-down in the hole (Fig. 5). In the subsequent stance phase, the ankle started to plantarflex earlier when walking through the hole than when walking on level ground. This resulted in greater synchronous plantarflexion and knee extension during the hole negotiation gait (Fig. 5). In the contralateral leg, the ankle joint was more dorsiflexed and the knee and hip joints were more flexed for most of the time during the hole negotiation gait (Fig. 5). The contralateral leg showed longer synchronous dorsiflexion and knee flexion during the second part of the stance phase of the preparation step and longer synchronous plantarflexion, knee and hip extension during the stance phase of the recovery step in the hole negotiation compared to level walking (Fig. 5).

**Fig. 6:**
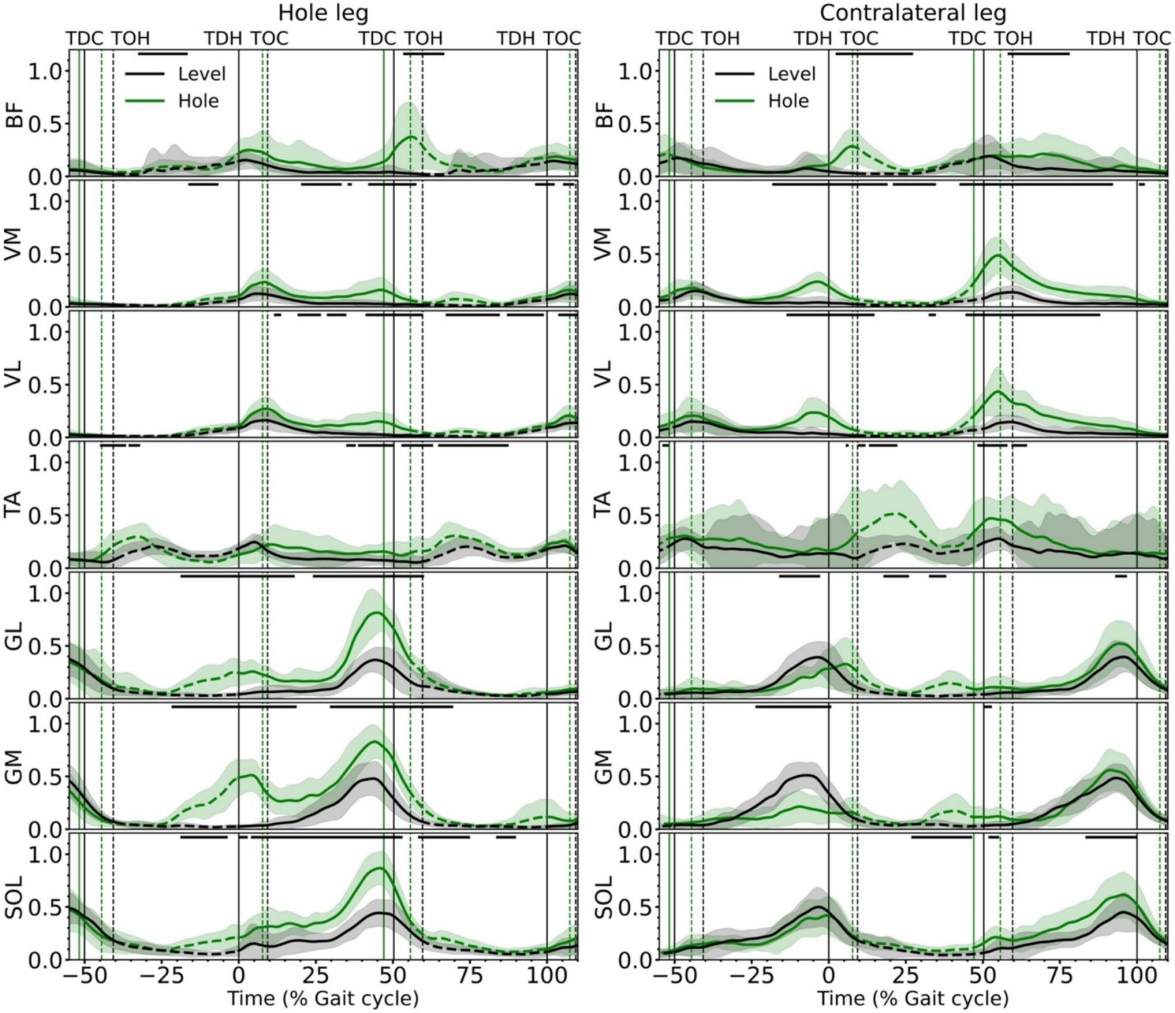
Activation patterns of the soleus (SOL), gastrocnemius medialis (GM), gastrocnemius lateralis (GL), tibialis anterior (TA), vastus lateralis (VL), vastus medialis (VM) and biceps femoris (BF) muscles of the hole (right) and contralateral (left) leg during level and hole negotiation walking. The curves and shaded areas represent mean ± standard deviation. The solid part of the curves indicates stance phase and the dashed part indicates swing phase. The horizontal axis is normalized to the gait cycle before touch-down in the hole (negative percentages) and to the gait cycle after touch-down in the hole (positive percentages). The vertical solid lines show the touch-down of the contralateral and the hole leg and the vertical dashed lines show the take-off. The horizontal black lines in the upper part of the figures define the areas of significant differences between level and hole negotiation (*p* < 0.05) based on the SPM analysis. *TDC* touch-down of the contralateral leg, *TOH* take-off of the hole leg, *TDH* touch-down of the hole leg, *TOC* take-off of the contralateral leg

The maximum activation of the vasti muscles was significantly higher in all three steps (preparation: *p* < 0.001, *d* = 1.21 and *p* < 0.001, *d* = 1.46; hole: *p* < 0.001, *d* = 1.48 and p < 0.001, *d* = 1.32; recovery: *p* < 0.001, *d* = 2.03 and *p* < 0.001, *d* = 3.06 for VL and VM) with the triceps surae muscles showing significantly higher values in the hole (SOL: *p* < 0.001, *d* = 2.73; GM: *p* < 0.001, *d* = 2.37; GL: *p* < 0.001, *d* = 2.66) and recovery steps (SOL: *p* < 0.001, *d* = 0.92; GM: *p* < 0.017, *d* = 0.02; GL: *p* < 0.001, *d* = 0.69) of the hole negotiation gait compared to level walking (Table 2). In the preparation step, the activation of the GM and GL of the contralateral leg, i.e. the leg in contact with the ground, was lower (*p* < 0.05) during the push-off phase of the hole negotiation gait (Fig. 6). The activation patterns of the vasti muscles of the contralateral leg in the preparation step are characterized by two maxima in their activation, one in the first part and the second in the second part of the stance phase during hole negotiation (Fig. 6). In level walking, the vasti showed only one maximum located in the first part of the stance phase (Fig. 6). During the stance phase in the hole, the triceps surae and vasti muscles of the hole leg are again characterized by two maxima in the hole negotiation gait compared to one maximum in level walking (Fig. 6). The second maximum was more pronounced than the first in the triceps surae muscles, and the first maximum was more pronounced than the second in the vasti muscles (Fig. 6).

**Table 2:** Maximum (max) activation of the soleus (SOL), gastrocnemius medialis (GM) and lateralis (GL), tibialis anterior (TA), vastus lateralis (VL) and vastus medialis (VM) and biceps femoris (BF) during stance of the preparation, in the hole and recovery steps in level and hole negotiation gait (mean ± standard deviation)

Variables	Preparation step		Step in the hole			Recovery step		
	Level walking	Hole negotiation	Level walking	Hole negotiation		Level walking		Hole negotiation
Activation_SOL,max_	0.54 ± 0.18	0.51 ± 0.15	0.52 ± 0.15	0.93 ± 0.15*	0.48 ± 0.18		0.67 ± 0.22*	
Activation_GM,max_	0.59 ± 0.12	0.31 ± 1.30*	0.55 ± 0.16	0.90 ± 0.13*	0.55 ± 0.12		0.62 ± 0.17*	
Activation_GL,max_	0.46 ± 0.18	0.43 ± 0.20	0.43 ± 0.14	0.88 ± 0.18*	0.44 ± 0.16		0.57 ± 0.21*	
Activation_TA,max_	0.36 ± 0.33	0.50 ± 0.40*	0.28 ± 0.06	0.32 ± 0.19	0.38 ± 0.32		0.58 ± 0.35*	
Activation_VL,max_	0.16 ± 0.10	0.33 ± 0.19*	0.17 ± 0.09	0.32 ± 0.10*	0.16 ± 0.07		0.47 ± 0.22*	
Activation_VM,max_	0.16 ± 0.08	0.29 ± 0.09*	0.14 ± 0.07	0.27 ± 0.13*	0.15 ± 0.06		0.53 ± 0.17*	
Activation_BF,max_	0.19 ± 0.15	0.33 ± 1.90*	0.16 ± 0.08	0.46 ± 0.31*	0.21 ± 0.19		0.28 ± 0.17*	

Note that the muscles of the contralateral leg are presented in the preparation and recovery steps, and the muscles of the hole leg are presented in the hole step

*Statistically significant difference to level walking (*p* < 0.05)

**Fig. 7:**
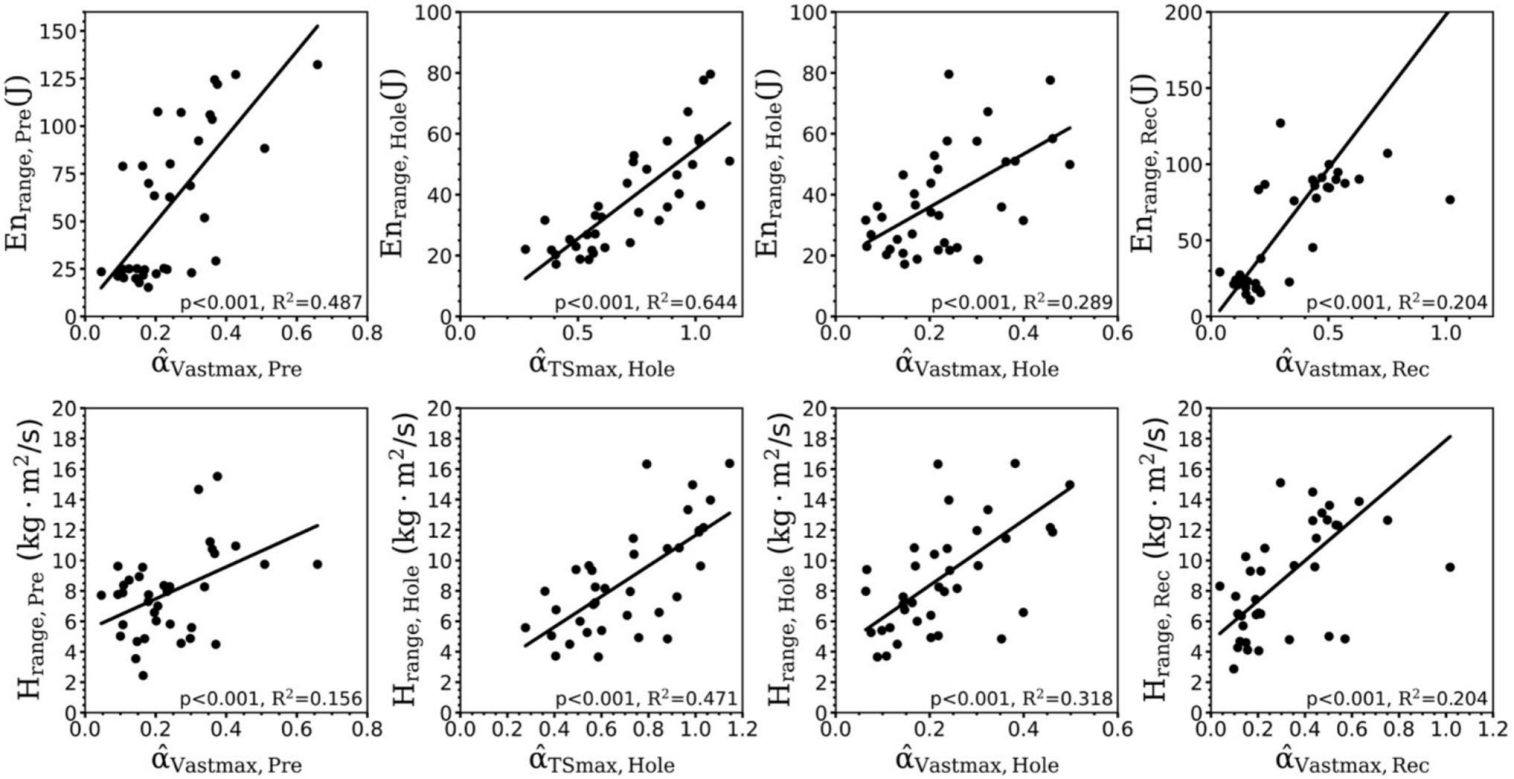
The first row shows the relationships of the range of the total centre of mass energy (En_range_) and the second row shows the relationships of the range of the frontal plane angular momentum (H_range_) during the stance phase of the preparation (Pre), hole (Hole) and recovery (Rec) steps with the maximum weighted activation of the vasti (α_vastmax_) and triceps surae (α_TSmax_) muscles of the stance leg

The second peak of the weighted activation of the vasti muscles of the contralateral leg was significantly related to the range of total CoM energy and the range of frontal plane angular momentum (both *p* < 0.001, Fig. 7) during the stance phase of the preparation step. Furthermore, the maximum weighted activation of the hole leg of both triceps surae and vasti muscles was significantly related to the range of total CoM energy and frontal plane angular momentum during the stance phase in the hole (in all cases *p* < 0.001, Fig. 7). Finally, during the stance phase of the recovery step, the maximum weighted activation of the vasti muscles was significantly related to the range of the total CoM energy and to the range of the frontal plane angular momentum (in both cases *p* < 0.001, Fig. 7).

## Discussion

For the hole negotiation gait, we found an increase in the peak-to-peak range of total CoM energy and whole-body angular momentum in the frontal plane during the stance phase of the preparation, hole and recovery steps, and a decrease in mediolateral MoS at touch-down of the preparation and hole steps. These results provide evidence for an increased challenge in the management of body energy, angular momentum and MoS during the hole negotiation gait. We also found anticipatory adjustments in CoM trajectories, joint angle kinematics and muscle activation patterns in both legs during hole negotiation gait that contribute to effective management of total CoM energy. In addition, anticipatory adjustments in the mediolateral leg angle during the preparation and hole steps, which reduced the mediolateral moment arm of the vertical ground reaction force during the subsequent stance phase, indicate simultaneous regulation of mechanisms that effectively control the management of frontal plane angular momentum. The results, however, also show that the anticipatory adjustments that effectively contribute to the required management of total CoM energy during hole negotiation imposes a challenge on the management of frontal plane angular momentum, and that the anticipatory adjustments in leg posture that favour the control of frontal plane angular momentum in turn compromise mediolateral MoS. The opposing effects of these adjustments on the control of total CoM energy, frontal plane whole-body angular momentum and mediolateral MoS, demonstrate trade-offs in the regulatory mechanisms for body stability and illustrate the challenge of moving on uneven terrain.

In the preparation step, the main strategy for negotiating the hole was to reduce the total CoM energy by absorbing energy before touch-down in the hole. The more dorsiflexed ankle and flexed knee joint angles of the contralateral leg during the preparation step induce a lowering of the CoM, which has also been reported in the past [24, 29, 51]. The reduced potential CoM energy due to the lowering of the body was not converted into kinetic energy but was absorbed by the human musculoskeletal system as the kinetic CoM energy remained similar to that of level walking. A second maximum in activation of the vasti muscles was accompanied by the observed decrease in potential CoM energy in the preparation step, and this maximum was associated with the range of total CoM energy during the stance phase. The significant relationship found between the maximum weighted activation of the vasti and the range of total CoM energy shows a significant involvement of the knee extensors in the absorption of energy. Furthermore, during the lowering phase of the CoM, there was a prolonged synchronous dorsiflexion and knee flexion, i.e. in-phase fluctuations in the two joints of the contralateral leg. The synchronous dorsiflexion and knee flexion in combination with the activated gastrocnemii suggests an energy transfer from the ankle to the knee joint via the biarticular gastrocnemii muscles [52]. This is an additional mechanism to absorb CoM energy by the voluminous quadriceps muscles [47].

The hole leg also shows modulations in joint angle kinematics and activation patterns to manage the total CoM energy during the step in the hole. The more plantarflexed ankle joint and the increased activation of the triceps surae muscles before touch-down are important predictive adjustments that provide advantages in energy absorption [19, 24, 53]. The increased maximum activation of the vasti and triceps surae muscles during the stance phase in the hole, compared to level walking, contributes to the management of total CoM energy. Both the maximum weighted activation of the vasti and triceps surae muscles of the hole leg were significantly related to the range of total CoM energy during the stance phase. In the recovery step, greater extension in the knee and hip joints, with greater activation of the vasti muscles of the recovery leg compared to level walking, was accompanied by an increase in total CoM energy during hole negotiation. We found a significant relationship between the maximum weighted activation of the vasti muscles and the range of total CoM energy during the stance phase of the recovery step, indicating that the greater muscle activation, together with the greater joint range of motion, contributed to the increase in total CoM energy by producing muscular work. The synchronous knee extension and plantarflexion of the hole and recovery legs indicate energy transfer from the knee to the ankle joint via the biarticular gastrocnemii muscles [52] during both the hole and recovery steps. In addition, the synchronous hip and knee extension of the recovery leg indicates energy transfer from the hip to the knee joint via the biarticular rectus femoris muscle during the recovery step of the hole negotiation gait. In both cases, there is a transfer of energy from the large proximal muscles to the ankle joint, which may increase ankle energy production through biarticular mechanisms [54–57]. All these observations show an increased involvement of mechanisms that effectively regulate the management of total CoM energy during the hole negotiation gait, starting from the preparation step and continuing to the recovery step.

Increased activation of the leg muscles is an important mechanism for the absorption and production of muscle energy and can support the management of total CoM energy. On the other hand, the vasti and triceps surae muscles act to rotate the body towards the contralateral leg [31] so the increased activation of these muscles required to manage the total CoM energy contributes to the increased range and rate of whole-body angular momentum in the frontal plane during the hole negotiation gait trials. The range of frontal plane angular momentum during hole negotiation was 36%, 70% and 97% greater than the range of frontal plane angular momentum of level walking during the stance phases of the preparation, hole and recovery steps, respectively. The rate of frontal plane angular momentum during the single stance phase of the hole and recovery steps was 72% and 70% higher, respectively, in hole negotiation. We found significant relationships between the range of frontal plane whole-body angular momentum and the maximum weighted activation of the vasti muscles during the lowering phase of the preparation step. Similarly, significant associations were found between the weighted maxima of vasti and triceps surae muscle activation and the range of frontal plane whole-body angular momentum in the hole step. Significant associations were also found between the weighted maximum activation of the vasti muscles and the range of frontal plane whole-body angular momentum during the recovery step. These results show that the increased muscle activation required to effectively manage the total CoM energy likely contributes to a concurrent increase in frontal plane angular momentum, thus challenging the control and regulation of frontal plane rotational body behaviour.

All participants were familiarized with hole negotiation by performing at least 4 trials prior to the trial of interest and had visual information about the location of the hole during the experiment. We found experience-based and visually guided anticipatory modulations of leg posture and step width during the hole negotiation gait. In the preparation and hole steps, the mediolateral leg angle at touch-down was 13% and 33% lower, respectively, i.e. closer to the vertical axis in the hole negotiation. The step width was 12% smaller during the hole step compared to level walking. The narrower step width and leg angle closer to the vertical axis in the frontal plane indicate a foot placement strategy that reduces the mediolateral moment arm from the CoM of the body to the vertical component of the ground reaction force vector. The decrease in the mediolateral moment arm is an important mechanism that causes a reduction in the moment of the vertical component of the ground reaction force, which rotates the body towards the contralateral leg [33]. On the other hand, a foot placement that positions the leg angle closer to the vertical axis decreases the mediolateral MoS, i.e. the extrapolated CoM moved closer to the boundary of the BoS [35]. A very small mediolateral MoS at the beginning of the stance phase can challenge body stability and may require corrective crossover steps during walking. Conversely, a large mediolateral MoS at the beginning of the stance phase can also challenge body stability by accelerating the CoM towards the contralateral side, which may increase the range of mediolateral body movement. Indeed, we found a reduction in the mediolateral moment arm of the vertical ground reaction force and MoS in the preparation and hole steps during the hole negotiation gait, indicating a trade-off between mechanisms that regulate the rotational and translational behaviour of the body. This anticipatory foot placement strategy shows that during hole negotiation gait, the modulation of mechanisms that control whole-body angular momentum in the frontal plane, i.e. the rotational behaviour of the body, is favoured over mechanisms that control MoS in the mediolateral direction, i.e. the translational behaviour of the body, possibly due to the greater challenge and need to regulate frontal plane angular momentum. Despite the decrease in the mediolateral moment arm of the vertical component of ground reaction force, peak-to-peak range of frontal plane angular momentum was higher during the stance phase of both preparation and hole steps during hole negotiation compared to level walking. Mechanically, the changes in whole-body angular momentum depend not only on the size of the generated moment around the CoM but also on the time interval over which it acts (i.e. angular impulse). The stance time of the preparation and hole step did not differ between level walking and hole negotiation gait, indicating that the greater peak-to-peak range of frontal plane whole-body angular momentum during hole negotiation was due to the amount of moment generated in the frontal plane. Therefore, we can argue that the high activation of the triceps surae and vasti muscles required to manage the total CoM energy during hole negotiation had a greater influence on frontal plane angular momentum than the aforementioned anticipatory adjustments to the moment arm and resulted in the increased range and rate of frontal plane angular momentum. In the recovery step, the stance time was increased in the hole negotiation gait and may contribute to the increased peak-to-peak range of frontal plane whole-body angular momentum during the recovery step.

In the current study, we used a 15 cm step-down hole, which is challenging for walking. Lower step-down hole heights reduce the consequences of hole negotiation in terms of reduced muscle activation and angular joint range of motion [29] and may lead to different negotiation strategies. Nevertheless, lowering the CoM in the preparation step as well as joint angle adjustments and increased muscle activation [19, 29, 30] are common strategies observed at different hole negotiation heights [24, 29, 51]. We determined the mediolateral moment arm of the vertical ground reaction force vector as the mediolateral distance between the CoM of the body and the CoM of the foot using the coordinates of the mediolateral axis of the global coordinate system rather than the distance between the CoM of the body and the centre of pressure. Possible shifts in the centre of pressure to the medial or lateral direction due to the ankle strategy [35] may affect the moment arms reported in the study. The leg angle in the mediolateral direction and step width showed clear changes in hole negotiation compared to level walking due to an anticipatory foot placement strategy, and considering that the ankle strategy shows a small, i.e. less than 2 cm, possible shift in the centre of pressure displacement [35], we are confident that the potential influence does not affect our main findings and conclusions. Finally, in the current study, we investigated young, healthy participants and identified the aforementioned trade-offs during hole negotiation gait. Older participants or individuals with pathologies may exhibit different behaviour, favouring larger safety margins over energy economy, for example, and thus employing different mechanisms to control frontal plane whole-body angular momentum, total CoM energy and mediolateral MoS. However, the aforementioned trade-offs that demonstrate the challenges of moving on uneven terrain would remain.

In conclusion, during hole negotiation gait in humans, (a) the challenge of managing total CoM energy, frontal plane angular momentum and mediolateral MoS is increased compared to level walking, despite anticipatory adjustments in mechanisms that control hole negotiation, (b) anticipatory adjustments in CoM trajectories, joint kinematic and muscle activation patterns in both legs regulate mechanisms that primarily support the management of total CoM energy and (c) the mutual interactions between the regulatory mechanisms for the management of total CoM energy, frontal plane angular momentum and mediolateral MoS demonstrate functional trade-offs, which are rooted in the nature of hole negotiation. Thus, the main message of the current study is that in order to effectively manage total CoM energy during hole negotiation, humans use mechanisms that lead to an increase challenge in the management of frontal plane angular momentum, i.e. effective control of total CoM energy at the expense of frontal plane angular momentum, and that adjustments in leg posture for advantageous control of frontal plane angular momentum compromise mediolateral MoS, trade-offs that demonstrate the challenge of moving on uneven terrain. The findings provide novel insights into the translational and rotational behaviour of the whole-body, how humans control body energy, frontal plane angular momentum and mediolateral MoS to maintain balance during hole negotiation. These insights are important with regard to the conceptualization of prevention and rehabilitation treatments aiming to improve stability control in healthy and pathological individuals, for the design of prostheses and exoskeletons for stable movement on uneven terrain and for the control of bipedal robots.

## Data Availability

10.6084/m9.figshare.26717926.
